# Rectal Microbiome Reveals the Improved Effect of Dietary Selenium Levels on Lactation Performance and Milk Fatty Acid Profiles in Lactating Donkeys

**DOI:** 10.3390/ani15223309

**Published:** 2025-11-17

**Authors:** Fanzhu Meng, Yanli Zhao, Yongmei Guo, Xiaoyu Guo, Qingyue Zhang, Zefu Wang, Li Li, Fang Hui, Manman Tong, Sumei Yan

**Affiliations:** Inner Mongolia Key Laboratory of Animal Nutrition and Feed Science, College of Animal Science, Inner Mongolia Agricultural University, Hohhot 010018, China; fzmeng2023@163.com (F.M.); ylzhao2010@163.com (Y.Z.); ymguo2015@163.com (Y.G.); gxy_2594@163.com (X.G.); alicezqy@126.com (Q.Z.); 15848152624@163.com (Z.W.); lily972021@163.com (L.L.); cf18447051697@163.com (F.H.); nndtmm@163.com (M.T.)

**Keywords:** selenium, lactating donkeys, lactation performance, fatty acid profiles, rectal flora structure

## Abstract

This study evaluated the effects of dietary selenium (Se) supplementation on lactation performance, milk fatty acid composition, and rectal microbiota and short-chain fatty acids in lactating Dezhou donkeys to determine the optimal dose. Twenty-four lactating Dezhou donkeys (estimated milk yield: 3.60 ± 0.84 kg/d; days in milk: 39.93 ± 7.02 d; body weight: 247.24 ± 26.27 kg; parity: 2.82 ± 0.48) were randomly divided into four groups (*n* = 6): CON (0 mg Se/kg DM), Se1 (0.15 mg Se/kg DM), Se2 (0.3 mg Se/kg DM), and Se3 (0.5 mg Se/kg DM), and were supplemented with Se yeast in the basal diet. The results showed that plasma Se concentrations increased dose-dependently. The Se2 group significantly increased milk yield, milk component yield, milk protein production efficiency, milk production efficiency, the digestibility of acid detergent fiber and neutral detergent fiber, protein biological value, and nitrogen metabolic rate, as well as the proportions of C18:1c9, C18:2c6, unsaturated fatty acids, monounsaturated fatty acids, polyunsaturated fatty acids, n-3 long-chain polyunsaturated fatty acids, the ratio of unsaturated to saturated fatty acids, the ratio of polyunsaturated to saturated fatty acids (P/S), desirable fatty acids, and (C18:0 + C18:1)/C16:0 in donkey milk, while decreasing the proportion of saturated fatty acids, atherogenicity index, and thrombogenic index. The Se2 group also increased acetate and butyrate in rectal feces, and promoted the growth of beneficial bacteria, including *Christensenellaceae R-7 group*, *Lachnospiraceae XPB1014*, and *norank_f_Erysipelotrichaceae*. In summary, the effects of Se supplementation were dose-dependent: 0.3 mg Se/kg DM improved milk performance and optimized milk fatty acid profiles by improving nutrient digestion and metabolism and modulating rectal bacteria structure, whereas 0.5 mg Se/kg DM showed no lactation benefits (with potential inhibitory effects) despite improving the P/S ratio and reducing the atherogenicity and thrombogenic indices.

## Simple Summary

Donkey milk is a highly nutritious food with recognized health benefits; however, its production is limited due to the low daily milk yield of donkeys. In this study, we investigated whether dietary supplementation with different doses of selenium (Se) could improve both the quantity and quality of milk in lactating donkeys. We found that the addition of 0.3 mg Se/kg DM significantly increased milk production and improved nutritional quality by elevating the content of beneficial fatty acids. This dosage also promoted the growth of beneficial gut bacteria and improved nutrient digestibility. In contrast, higher Se doses did not improve milk yield and may potentially suppress it, with an increase in the polyunsaturated to saturated fatty acid (P/S) ratio and a reduction in the atherogenicity and thrombogenicity indices. These findings provide practical guidance for the safe use of Se in donkey feeding strategies, facilitating more sustainable dairy production and yielding healthier milk for consumers.

## 1. Introduction

With continuous improvements in living standards and health awareness, consumer demand for high-quality, healthy food is growing. Donkey milk has attracted increasing attention as a functional food with potential benefits for human health [1]. Characterized by its low fat content and high levels of n-3 polyunsaturated fatty acids (n-3 PUFA) [2], donkey milk may be particularly suitable for individuals with cardiovascular diseases. Owing to its palatability and low levels of caseins and other allergenic proteins, it is also a suitable nutritional source for children with a cow’s milk protein allergy [3,4]. Furthermore, extensive research confirms that donkey milk possesses diverse bioactive properties, including antimicrobial [5], anticancer [6], antioxidant [7], immunomodulatory [8], and hypoallergenic activities [9]. These functional attributes underscore its potential in health promotion and disease prevention [10,11]. However, donkeys have a low milk yield, producing only about 3 kg per day [1]. Therefore, improving milk yield and optimizing the FA composition of donkey milk are of great significance.

Selenium (Se) is an essential trace element that exerts diverse, dose-dependent physiological functions, including the improvement of lactation performance, antioxidant capacity, immunity, growth, and meat quality [12,13,14]. To date, limited research has been conducted on the effects of dietary Se supplementation on lactation performance. A study by Sun et al. [15] on dairy cows showed that increasing Se supplementation induced quadratic improvements in the yield of raw milk, protein, and lactose. Se supplementation in feed typically uses additives like sodium selenite, sodium selenate, and selenium yeast. Compared to inorganic forms (e.g., sodium selenite and sodium selenate), organic selenium (such as selenomethionine and selenium yeast) exhibits higher absorption and utilization efficiencies [16]. Tong et al. [14] demonstrated that dietary Se supplementation not only effectively elevates Se levels in milk and blood but also improves lactation performance, antioxidant capacity, and immune function in lactating donkeys. Sun et al. [17] revealed that dietary supplementation with 5 mg Se/kg DM significantly reduced milk somatic cell count (SCC). Furthermore, serum Se concentrations increased dose-dependently with elevated dietary Se levels. These findings indicate that supplementation with an appropriate Se dose could effectively improve udder health and Se status in donkeys. Nevertheless, relevant research remains limited, and the underlying mechanisms are not yet fully understood.

The gut microbiota is integral to the nutritional physiology of monogastric animals, primarily owing to its direct functional association with dietary utilization [18]. Furthermore, these microbial communities play a determinant role in optimizing livestock production efficiency through their metabolic interactions with the host’s digestive system [19]. Donkeys are monogastric herbivores that rely on hindgut fermentation. The enlarged hindgut, primarily composed of the cecum and colon, enables them to extract energy and nutrients from fibrous feeds through microbial fermentation [20]. Considering the challenges of directly accessing these intestinal regions for content collection and the fact that fecal samples can represent the gut microbiota [21], we utilized fecal samples to assess the influence of Se on the gut microbiota of lactating donkeys, given that fecal samples harbor the majority of cecal microbiota and mirror the bacterial composition in the cecum [22,23]. Although the composition and function of the gut microbiota are known to affect nutrient digestibility and lactation outcomes in donkeys [24,25], the impact of dietary Se on these microbial communities in lactating jennies remains poorly understood. While studies in ruminants such as sika deer have shown that Se modulates bacterial abundance and enhances fiber degradation [26,27], comparable research in donkeys is limited. Therefore, this study aimed to investigate the effects of dietary Se supplementation on lactation performance, milk FA profiles, and rectal microbiota in lactating donkeys, and to determine the optimal Se dose, providing valuable insights for the strategic management of lactating donkey production.

## 2. Materials and Methods

The experiment was conducted in Inner Mongolia Grassland Yulv Science and Technology Animal Husbandry Co., Ltd. (Hohhot, China). The Animal Ethics and Welfare Committee approved the experimental procedures at the Inner Mongolia Agricultural University (NND2021050), which were under the university’s guidelines for animal research.

### 2.1. Animals, Diets, and Experiment Design

In a single factor completely randomized experimental design, twenty-four healthy lactating Dezhou donkeys (estimated milk yield: 3.60 ± 0.84 kg/d; days in milk: 39.93 ± 7.02 d; body weight: 247.24 ± 26.27 kg; parity: 2.82 ± 0.48) were randomly assigned to four groups (*n* = 6): a control (CON) group fed a basal diet, and three treatment groups (Se1, Se2, Se3) supplemented with selenium yeast to provide 0.15, 0.3, and 0.5 mg Se/kg DM, respectively. The basal diet (concentrate-to-forage ratio 30:70; Table 1), prepared as a single batch with a background Se level of 0.04 mg/kg DM, was used throughout the experiment. The selenium yeast product (SelenoSource AFTM2000, Diamond V Biological Fermentation Engineering & Technologies Shenzhen Co., Ltd., Shenzhen, China) contained 0.2% (2000 mg/kg) total Se, of which ≥98% was organic Se primarily in the form of selenomethionine. The experiment lasted 10 weeks, comprising a 2-week adaptation period and an 8-week experimental period for data and sample collection.

To ensure precise measurement of individual feed intake and avoid interference from social behaviors on the experimental results, the donkeys were housed in individual pens. These pens allowed for visual, auditory, and olfactory contact to meet animals’ social needs. The donkeys were kept in individual stalls (1.6 m × 2.0 m) and fed a diet of concentrate, corn silage, and alfalfa twice daily (at 07:00 and 14:00). Millet straw was offered five times daily, and water was available ad libitum. Daily feed allowances were adjusted based on the previous day’s intake to maintain a refusal rate of 5~10%. Any unconsumed feed was collected before morning feeding, and the concentrate-to-forage ratio was strictly maintained throughout the experiment.

### 2.2. Milk Sampling and Analysis

Milk yield was recorded daily over seven consecutive days at each biweekly sampling point (weeks 2, 4, 6, and 8). Donkeys were separated from their foals for two 3 h periods daily (07:00–10:00 and 14:00–17:00) to allow for milk accumulation. Milking was performed at 10:00 and 17:00 each day using an individual vacuum pump (JuduH5402, Judu Technology, Xingtai, China) operating at a vacuum level of 50 kPa, a pulse frequency of 60 cycles per minute, and a pulsation ratio of 60:40 (suction phase to relief phase). The milk yield (MY, kg/day) from both sessions was measured using a lactometer integrated into the milking equipment and summed to obtain the total yield over the 6 h separation period. The estimated milk yield (EMY, kg/day) was then calculated as follows: EMY (kg/day) = MY (total milk yield over 6 h) (kg/day)/6 (h) × 24 (h) [28,29]. Daily weighing of the feed provided and refused was conducted to determine the dry matter intake (DMI) for each donkey. Milk production efficiency was calculated as described below. Additionally, during the final three days of each biweekly period, morning and afternoon milk samples were composited in a 1:1 ratio. One aliquot was treated with a preservative (D & F Control Systems Inc., Beverly, MA, USA) and underwent immediate compositional analysis for protein, fat, lactose, solids-not-fat (SNF), and total solids (TS) using a MilkoScan FT+ infrared analyzer (Foss Analytical, Hillerød, Denmark), while SCC was quantified using a Foss-somatic FC counter. Both instruments were calibrated specifically for donkey milk using reference samples with known compositions determined by standard chemical methods. The remaining aliquot was stored at −20 °C for fatty acid (FA) profiling. The following calculations were applied: solids-corrected milk (SCM, kg/day) = {(12.3 × milk fat (%) content of nonstandard milk + 6.56 × SNF (%) content of nonstandard milk − 0.0752)} × EMY (kg/day) [30]. Milk production efficiency = SCM (kg/day)/DMI (kg/day) × 100. Milk protein synthesis efficiency = {EMY (kg/day) × milk protein (%)}/{DMI (kg/day) × dietary crude protein (CP) content (%)} [31].

FA methyl esters (FAMEs) were produced from 1 g samples of freeze-dried donkey milk powder according to the methodology of O’Fallon et al. [32]. FA concentrations were determined using a gas chromatograph (Agilent 7890B, Agilent Technologies, Santa Clara, CA, USA) equipped with an SP-2560 capillary column (Supelco; 100 m × 0.25 mm, 0.2 μm film thickness). The injector temperature was set at 248 °C. The oven temperature program was as follows: initiate temperature of 120 °C held for 5 min; increased to 170 °C at 3 °C/min and held for 10 min; further increased to 220 °C at 3 °C/min and held for 5 min; finally increased to 240 °C at 1 °C/min and held for 10 min. Nitrogen was used as the carrier gas at a constant flow rate of 3 mL/min with a split ratio of 9:1. The FAs were identified by comparing the retention times of sample peaks with those of a known FAME standard mixture (CRM47885, Sigma-Aldrich, St. Louis, MO, USA). Quantification was performed using the external standard method, with calibration curves established for each FA; all curves demonstrated excellent linearity.

The contents of 37 individual FAs were determined. Based on these, the following parameters were calculated: saturated fatty acid (SFA), unsaturated fatty acid (UFA), monounsaturated fatty acid (MUFA), polyunsaturated fatty acid (PUFA), n-6 PUFA, n-3 PUFA, n-6 long-chain polyunsaturated fatty acids (n-6 LCPUFA), n-3 long-chain polyunsaturated fatty acids (n-3 LCPUFA), along with the ratios of n-6 PUFA/n-3 PUFA (n-6/n-3), UFA/SFA (U/S), and PUFA/SFA (P/S). Also calculated were the values for desirable fatty acid (DFA), atherogenicity index (AI), and thrombogenic index (TI). SFA in the milk included C4:0, C6:0, C8:0, C10:0, C11:0, C12:0, C13:0, C14:0, C15:0, C16:0, C17:0, C18:0, C20:0, C21:0, C22:0, C23:0, and C24:0. MUFA included C14:1, C15:1, C16:1, C17:1, C18:1t9, C18:1c9, C20:1, C22:1, and C24:1. n-6 PUFA included C18:2t6, C18:2c6, C18:3n6, C20:2n6, C20:3n6, C20:4n6, and C22:2n6. n-6 LCPUFA included C20:2n6, C20:3n6, C20:4n6, and C22:2n6. n-3 PUFA included C18:3n3, C20:3n3, C20:5n3, and C22:6n3. n-3 LCPUFA included C20:3n3, C20:5n3, and C22:6n3. The following formulas were applied: PUFA = n-3 PUFA + n-6 PUFA; DFA = C18:0 + UFA [33]; AI = (C12:0 + 4 × C14:0 + C16:0)/UFA [34]; TI = (C14:0 + C16:0 + C18:0)/[0.5 × MUFA) + 0.5 × n-6 PUFA + 3 × n-3 PUFA + (n-3 PUFA/n-6 PUFA)] [34]; and (C18:0 + C18:1)/C16:0 [33].

### 2.3. Apparent Nutrient Digestion and Metabolism

Before the experiment commenced, dietary samples were collected, oven-dried at 65 °C, homogenized by crushing, sieved, and stored in a dark, well-ventilated, and low-humidity environment for subsequent nutrient composition analysis. During the eighth week of the experiment, a daily sample of 200 g of rectal feces was collected from each donkey over six consecutive days. Fecal samples from individual donkeys were homogenized and split. One aliquot was treated with 10% H_2_SO_4_ (*v*/*v*) to minimize nitrogen (N) volatilization and subsequently stored at −20 °C pending N analysis. The remaining aliquot was oven-dried at 65 °C for the determination of dry matter (DM; method 930.15), CP (method 984.13), ether extract (EE; method 920.39), and acid-insoluble ash (AIA; method 975.12) based on AOAC International methods [35]. Neutral detergent fiber (NDF) and acid detergent fiber (ADF) were determined using an Ankom 220 Fiber Analyzer (Ankom Technology, Macedon, NY, USA) following Van Soest et al. [36]. Apparent total-tract digestibility (ATTD) was calculated using the AIA method as described by Ren et al. [37], with the following formula:

ATTD (%) = 100 − [(A1 × B)/(A × B1)] × 100.

where A = nutrient content in the diet (%), A1 = the same nutrient content in feces (%), B = dietary AIA content (%), and B1 = fecal AIA content (%).

A metabolic trial was conducted during the final six days of the experimental period, following the methods outlined by Liang et al. [29]. For urine collection, donkeys were temporarily placed in a metabolic cage each morning and afternoon. During each placement, a clean plastic bucket covered with gauze was positioned on the ground for urine collection. A minimum volume of 200 mL was obtained per collection. Samples were then transferred to specimen containers (Corning Costar, Corning Incorporated, Corning, NY, USA). One aliquot remained untreated for creatinine and gross energy (GE) quantification, while the other was acidified with 10 N sulfuric acid to fix nitrogen prior to urinary nitrogen analysis [38]. All samples were stored at −20 °C.

GE in the diet, feces, and urine was determined using an oxygen bomb calorimeter (Parr 6400 Automatic Analyzer, Parr Instrument Company, Moline, IL, USA) following Jha et al. [39]. Energy digestibility and metabolizability were computed using the following formulas: Energy metabolizability = {diet GE (MJ/kg) × DMI (kg) − fecal GE (MJ/kg) × fecal output (kg) − urine GE (MJ/kg) × urine output (kg)}/{diet GE (MJ/kg) × DMI (kg)};

Where fecal output (kg) = DMI (kg) × (AIA % in feed/AIA % in feces), urine output (kg) was calculated using a creatinine-based method [29]: urine output = body weight (kg) × 24.05/urinary creatinine/113; N metabolizability = protein biological value (BV) × N digestibility; BV = {N intake (kg/day) − feces N (kg/day) − urine N (kg/day)}/{N intake (kg/day) − feces N (kg/day)} × 100%.

### 2.4. Blood Sampling and Analysis

Blood samples were collected via jugular venipuncture into sodium heparin tubes (Corning, NY, USA) before the morning feeding (07:00) on two consecutive days during week 8. After centrifugation at 2500× *g* for 15 min, the plasma was separated and stored at −20 °C. The FA content in the plasma was determined using an Agilent 7890B gas chromatograph (Agilent Technologies, Wilmington, DE, USA) equipped with an SP-2560 capillary column (Supelco; 100 m × 0.25 mm, 0.2 μm film thickness), following the method described by Wang et al. [32]. The chromatographic parameters were consistent with those used for donkey milk analysis. The Se concentration in the plasma samples was determined according to the National Food Safety Standard of China (GB5009.268-2016 [40]) with modifications. Briefly, 1 mL of plasma was digested with 20–30 mL of a nitric acid:perchloric acid mixture (2:1, *v*/*v*) for 1 h or overnight. Subsequently, the samples were heated at 300 °C on an electric hot plate until the solution became clear or light yellow. After cooling, the digest was diluted to a predetermined volume with deionized water. The Se concentration was then measured using inductively coupled plasma optical emission spectrometry (ICP-OES; ICAP 6300Duo, Thermo Fisher Scientific, Waltham, MA, USA).

### 2.5. Short-Chain Fatty Acids Analysis of the Feces

On the final day of the experiment, rectal feces samples were collected from lactating donkeys using sterile gloves (two collections per donkey). The samples were stored in DNase- and RNase-free tubes (Shanghai Jingke Chemical Technology Co., Ltd., Shanghai, China), immediately frozen in liquid nitrogen (−196 °C), and stored for subsequent analysis of short-chain fatty acids (SCFAs). The SCFAs analyzed included acetate, propionate, iso-butyrate, butyrate, isovalerate, and valerate. SCFAs were extracted from 1.5 g of fecal samples following the method described by Li et al. [25]. Analysis was performed using a Shimadzu 2014 gas chromatograph (Shimadzu Corporation, Kyoto, Japan) equipped with a DB-FFAP column (60 m × 0.25 mm × 0.5 μm). Nitrogen was used as the carrier gas. The oven, detector, and injector temperatures were set at 120 °C, 250 °C, and 220 °C, respectively. Quantification was achieved by comparison with known standards (Supelco Volatile Fatty Acid Standard Mix, Sigma-Aldrich, St. Louis, MO, USA).

### 2.6. Rectal Microbiome Analysis

Total microbial DNA was extracted from thawed fecal samples using the E.Z.N.A. Soil DNA Kit (Omega Bio-tek, Norcross, GA, USA) according to the manufacturer’s protocol. DNA quality was assessed by measuring purity and concentration with a NanoDrop2000 spectrophotometer (Thermo Fisher Scientific, Wilmington, DE, USA) and verifying integrity by 1% agarose gel electrophoresis. The V3–V4 hypervariable region of the bacterial 16S rRNA genes was amplified with primers 338F (5′-ACTCCTACGGGAGGCAGCAG-3′) and 806R (5′-GGACTACHVGGGTWTCTAAT-3′) in 20 μL PCR reactions. The reaction mixture contained 4 μL of 5× FastPfu Buffer, 2 μL of 2.5 mM dNTPs, 0.8 μL each primer (5 μM), 0.4 μL of FastPfu Polymerase, 0.2 μL of BSA, and 10 ng template DNA. Amplification was performed using an ABI GeneAmp 9700 thermocycler (Applied Biosystems, Foster City, CA, USA). Amplicons from triplicate PCRs per sample were pooled, electrophoresed on 2% agarose gels, and purified using a PCR Clean-Up Kit (YuHua, Taizhou, China). After quantification with a Qubit 4.0 Fluorometer (Thermo Fisher Scientific), the purified amplicons were subjected to paired-end sequencing (2 × 300 bp) on Illumina MiSeq PE300 platform (Majorbio, Shanghai, China) following standardized protocols.

After initial processing with fastp (version 0.19.6), paired-end reads were merged using FLASH (version 1.2.7) with stringent parameters: truncating reads at sites with average quality <20 (50 bp window), discarding reads <50 bp or with ambiguous bases, assembling overlaps >10 bp (max mismatch 0.2), and matching barcodes/primers with exact barcode and ≤2 primer mismatches. Processed sequences were clustered into OTU at 97% similarity using UPARSE (version 7.1, http://drive5.com/uparse/, accessed on 17 June 2024). Taxonomic classification of representative OTU sequences was performed using the RDP Classifier (version 2.2) against the SILVA v138 16S rRNA database with a confidence threshold of 0.7. Subsequent analyses included alpha-diversity analysis (http://www.mothur.org/wiki/Calculators, accessed on 17 June 2024), construction of Venn diagrams, community composition analysis, intergroup significance testing at the phylum and genus levels, LEfSe analysis, and calculation of Spearman rank correlation coefficients between environmental factors and prominent genera. The resulting numerical matrices were visualized as heatmaps, where colors indicate the magnitude of values within the matrix. Beta diversity was assessed through principal coordinate analysis (PCoA) based on Bray–Curtis distances. All statistical analyses and visualizations were implemented in R. (version 3.3.1).

### 2.7. Statistical Analysis

All statistical analyses were performed using SAS software (version 8.1). The PROC MIXED procedure was applied to lactation performance and SCC using the model Yijkm = μ + Ci + Wj + Ci × Wj + bXjk + Sim + εijkm, where the terms are as follows: Yijkm (dependent variable), μ (overall mean), Ci (fixed effect of dietary Se level), Wj (fixed effect of lactation week: 2, 4, 6, 8), Ci × Wj (effect of the interaction between diet treatment and lactation week), bXjk (covariate from pretrial week 0), Sim (random effect of donkey), and εijkm (residual error). Nutrient digestibility, nitrogen and energy metabolism indicators, plasma Se concentration, FA composition in blood and milk, SCFA concentrations in rectal feces, and alpha-diversity indices were analyzed using the GLM procedure for normally distributed data; otherwise, the Kruskal–Wallis test was applied. Differences across treatments were analyzed using Duncan’s multiple range test, and data are reported as least-squares means along with standard errors. Differentially abundant bacterial genera in the rectal microbiota were identified using linear discriminant analysis effect size (LEfSe), with a significance threshold set at a logarithmic LDA score of >2.5. Spearman’s correlation analysis was performed to examine the associations between differentially abundant bacteria genera and SCFA concentrations, FA profiles, nutrient digestion and metabolism rates, and lactation performance. A *p*-value < 0.05 was considered statistically significant, while 0.05 ≤ *p*-value < 0.10 was considered a trend toward significance.

## 3. Results

### 3.1. Lactation Performance

As shown in Table 2, dietary Se supplementation significantly affected DMI, lactation performance, and SCC in lactating donkeys. Compared with the CON group, donkeys in the Se2 group had significantly greater (*p* < 0.05) EMY, milk production efficiency, milk protein synthesis efficiency, and yields of lactose, SNF, and TS. The fat yield was significantly improved (*p* = 0.008) in the Se1, Se2, and Se3 groups compared to the CON group. The protein yield in the Se2 group demonstrated an increasing trend compared to both the CON and Se1 groups (*p* = 0.063). Compared with the CON group, SCC in the Se2 and Se3 groups was significantly decreased (*p* = 0.019).

### 3.2. Nutrient Digestibility

As shown in Table 3, compared to the CON group, the apparent digestibility of ADF was significantly increased in the Se2 and Se3 groups (*p* = 0.036), while the NDF in the Se2 and Se3 groups showed a tendency to increase compared to that in the CON group (*p* = 0.064). The protein biological value and nitrogen metabolic rate in the Se2 group were significantly higher than those in the CON, Se1, and Se3 groups (*p* = 0.019; *p* = 0.012).

### 3.3. Plasma Selenium Concentration and Fatty Acid Composition in Plasma and Milk

As presented in Table 4, the concentration of plasma Se in the Se1, Se2, and Se3 groups was significantly increased (*p* < 0.0001) compared to the CON group. The concentration in the Se3 group was also significantly greater than that in the Se1 group, and no significant differences were observed between the Se2 group and either the Se1 or Se3 groups. Compared to the CON group, the proportions of C17:0, C15:1, and C22:1 in the Se1, Se2, and Se3 groups were significantly increased (*p* = 0.046; *p* < 0.0001; *p* < 0.0001), while the proportions of C20:1 were opposite (*p* = 0.005). The proportions of C4:0, C6:0, C8:0, C11:0, C17:1, UFA, MUFA, n-3 LCPUFA, U/S, and P/S in the Se2 and Se3 groups were significantly increased (*p* < 0.05). Conversely, the proportions of C18:0 and SFA were significantly decreased (*p* = 0.007; *p* = 0.011). The proportions of C22:0, C22:2n6, C20:3n3, and C20:5n3 in the Se2 group were significantly increased (*p* < 0.05). Conversely, the proportions of C13:0, C18:2t6, C18:3n6, and TI were significantly decreased (*p* < 0.05). DFA in the Se2 group showed a greater tendency to increase than that in the CON group (*p* = 0.079). The proportion of C21:0 in the Se1 and Se3 groups were significantly increased (*p* < 0.0001). The proportion of C22:6n3 in the Se3 group showed a greater tendency to increase than that in CON group (*p* = 0.089). Compared with the CON group, the proportion of C10:0 in the Se3 group was significantly increased, and that in the Se2 group was significantly decreased (*p* < 0.0001).

The effects of different levels of Se in the diet on the milk FA composition of lactating donkeys are presented in Table 5. Compared to the CON group, the proportions of C6:0, C13:0, C16:1, C18:1c9, C22:2n6, C20:3n3, UFA, MUFA, n-3 LCPUFA, U/S, DFA, and (C18:0 + C18:1)/C16:0 in the Se1, Se2, and Se3 groups were significantly increased (*p* < 0.05), while the proportions of C18:1t9, C22:1, SFA, and TI were opposite (*p* < 0.05). The proportions of C11:0, C18:2c6, PUFA, and P/S in the Se2 and Se3 groups were significantly increased (*p* < 0.05). Conversely, the proportions of C16:0, C20:0, and AI exhibited a significant decrease (*p* = 0.002; *p* < 0.001; *p* < 0.001), while the proportion of C18:0 demonstrated a declining trend (*p* = 0.065). The proportions of C4:0, C8:0, and C10:0 in the Se2 group were significantly increased (*p* = 0.003; *p* = 0.034; *p* = 0.001). The proportions of C14:1, C18:3n3, n-3 PUFA, and n-6 PUFA in the Se3 group were significantly increased (*p* < 0.05). Conversely, the proportion of C12:0 was significantly decreased (*p* = 0.023). Compared with the CON group, the proportion of C15:0 in the Se1 and Se3 groups was significantly increased, and that in the Se2 group was significantly decreased (*p* < 0.001). Compared to the Se1 group, the proportions of C20:1 in the Se3 group had a tendency to decrease (*p* = 0.080). Compared to the Se1 and Se3 groups, the proportions of C20:2n6 and n-6 LCPUFA in the Se2 group were significantly decreased (*p* = 0.018).

The effects of different levels of Se in the diet on the milk FA yield of lactating donkeys are presented in Table 6. Compared to the CON group, the proportions of C6:0, C13:0, C20:4n6, C22:2n6, U/S, and (C18:0 + C18:1)/C16:0 in the Se1, Se2, and Se3 groups were significantly increased (*p* < 0.05). Conversely, the proportions of C22:1 and TI were significantly decreased (*p* = 0.004; *p* < 0.0001). Additionally, there was a trend toward an increase in the proportion of C20:3n6 (*p* = 0.058). The proportions of C8:0, C10:0, C16:1, C18:1c9, C20:3n3, UFA, and n-3 LCPUFA in the Se1 and Se2 groups were significantly increased (*p* < 0.05), while the proportions of C18:1t9 were opposite (*p* < 0.0001). The proportions of C11:0, C18:3n3, PUFA, n-3PUFA, and P/S in the Se2 and Se3 groups were significantly increased (*p* < 0.05). Conversely, the proportions of AI exhibited a significant decrease (*p* < 0.001). The proportions of C4:0, C18:2c6, UFA, and DFA in the Se2 group were significantly increased (*p* < 0.05), while the proportion of n-6PUFA demonstrated an increasing trend (*p* = 0.056). The proportions of C12:0, C16:0, and SFA in the Se3 group were significantly decreased (*p* = 0.005; *p* = 0.006; *p* = 0.001). Compared to the Se1 and Se2 groups, the proportions of C20:1 in the Se3 group were significantly decreased (*p* = 0.022).

### 3.4. Short-Chain Fatty Acids in the Rectal Feces

As shown in Table 7, acetate, butyrate, isovalerate, and acetate-to-propionate ratio increased in the Se2 and Se3 groups compared with the CON group (*p* < 0.05). The total volatile fatty acid (Total VFA) level in the Se3 group was increased compared with the CON group (*p* = 0.046). There were no significant differences in propionate, isobutyrate, and valerate concentrations between the CON and AOE groups (*p* > 0.05).

### 3.5. Fecal Bacterial Richness, Diversity, and Composition

**Figure 1:**
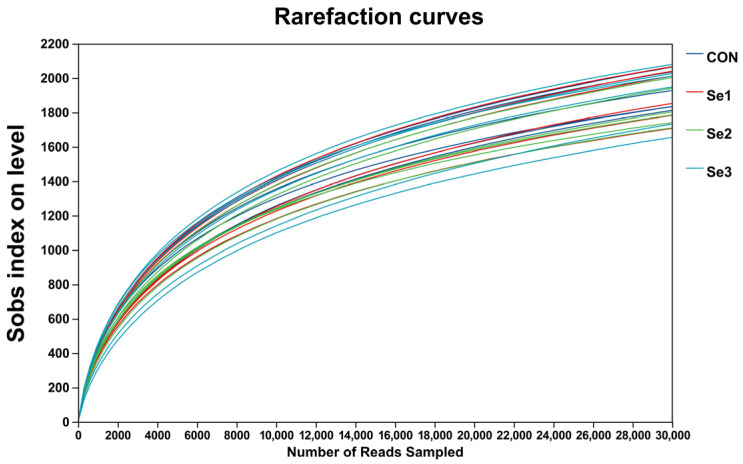
Observed OTU (Sobs) rarefaction curves of rectal bacteria across selenium supplementation groups. CON = supplemented with 0 mg of Se/kg DM, Se1 = supplemented with 0.15 mg of Se/kg DM, Se2 = supplemented with 0.3 mg of Se/kg DM, Se3 = supplemented with 0.5 mg of Se/kg DM.

**Figure 2:**
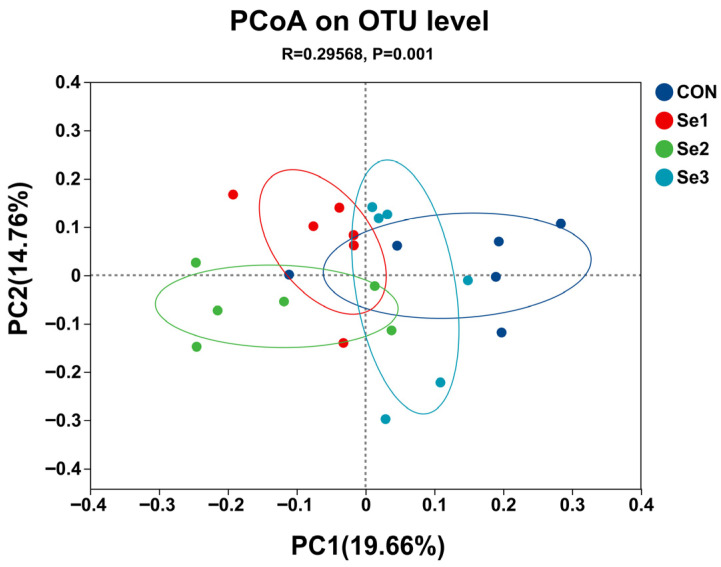
Principal coordinate analysis (PCoA) of rectal bacteria composition at genus level across selenium supplementation groups. CON = supplemented with 0 mg of Se/kg DM, Se1 = supplemented with 0.15 mg of Se/kg DM, Se2 = supplemented with 0.3 mg of Se/kg DM, Se3 = supplemented with 0.5 mg of Se/kg DM.

**Figure 3:**
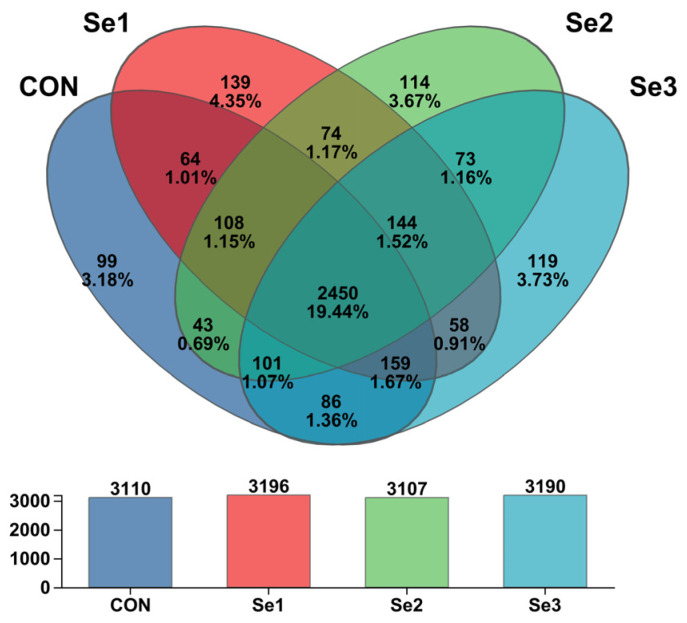
Venn analysis graph of rectum bacteria at OTU level across selenium supplementation groups. CON = supplemented with 0 mg of Se/kg DM, Se1 = supplemented with 0.15 mg of Se/kg DM, Se2 = supplemented with 0.3 mg of Se/kg DM, Se3 = supplemented with 0.5 mg of Se/kg DM.

The rarefaction curves of Sobs reached a plateau (Figure 1), indicating that the sequencing depth in this study was adequate to capture the diversity and structure of the rectal microbiota. The α-diversity analysis revealed that there were no differences (*p* > 0.05) in the Sobs, Shannon, Simpson, Ace, and Chao1 indices among the treatment groups. The coverage index for each group reached 0.98, demonstrating that the sequencing depth was adequate for subsequent analysis (Table 8). The PCoA revealed a clear separation of the rectal microbiota at the genus level among the four treatment groups (Figure 2), indicating that Se intake levels significantly influenced microbial community structure. Non-repeat sequences were clustered into OTUs at a 97% similarity threshold, yielding 2921 OTUs in total. Among these, 99, 139, 114, and 119 unique OTUs were identified in the CON, Se1, Se2, and Se3 groups, respectively (Figure 3).

### 3.6. Significantly Different Rectum Bacteria Among the CON, Se1, Se2, and Se3 Groups

**Figure 4:**
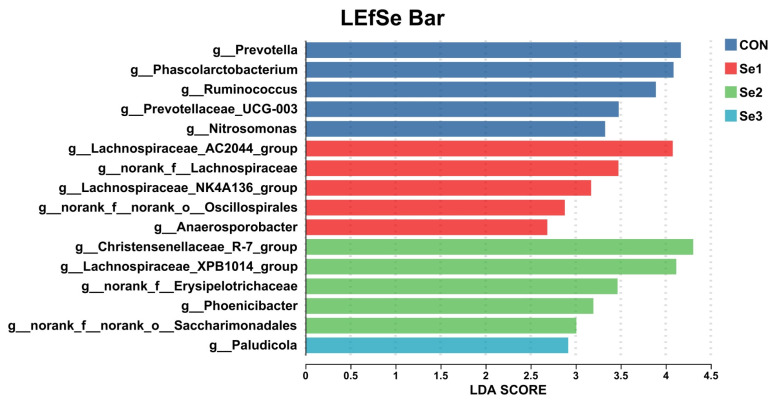
Effects of Se supplementation on the composition of rectal bacteria at the genus level. LDA scoreS > 2.5 were defined as significant difference. CON = supplemented with 0 mg of Se/kg DM, Se1 = supplemented with 0.15 mg of Se/kg DM, Se2 = supplemented with 0.3 mg of Se/kg DM, Se3 = supplemented with 0.5 mg of Se/kg DM.

To elucidate the effects of different Se intake levels on the rectal bacterial structure of donkeys, we analyzed the bacterial changes at the phylum and genus levels across the four treatment groups. At the phylum level, the rectal microbiota was predominantly composed of *Firmicutes*, *Bacteroidota*, *Spirochaetota*, *Verrucomicrobiota*, and *Actinobacteriota* (Table 9). Compared with the Se1 group, the Se2 group increased the relative abundance of *Actinobacteriota* (*p* < 0.05). No significant changes were found between the CON, Se3, and the other groups. LEfSe analysis identified bacterial taxa with significantly different abundances among the four groups (Figure 4). The relative abundances of *Lachnospiraceae_AC2044_group*, *Anaerosporobacter, norank_f__Lachnospiraceae*, and *norank_f__norank_o__Oscillospirales* were significantly higher in the Se1 group than in the CON, Se2 and Se3 groups. In the Se2 group, the relative abundances of *Christensenellaceae_R-7_group*, *Lachnospiraceae_XPB1014*, *norank_f_Erysipelotrichaceae*, *Phoenicibacter*, and *norank_f_norank_o_Saccharimonadales* were significantly increased. The Se3 group showed an increased relative abundance of *Paludicola*. In contrast, Se supplementation decreased the relative abundances of *Prevotella*, *Phascolarctobacterium*, *Ruminococcus*, *Prevotellaceae_UCG-003*, and *Nitrosomonas* in the rectum.

### 3.7. Correlation Analysis of Differential RECTAL Bacteria Genus with Nutrient Digestion and Metabolism Rate, Lactation Performance, Milk FA Composition, and SCFA

**Figure 5:**
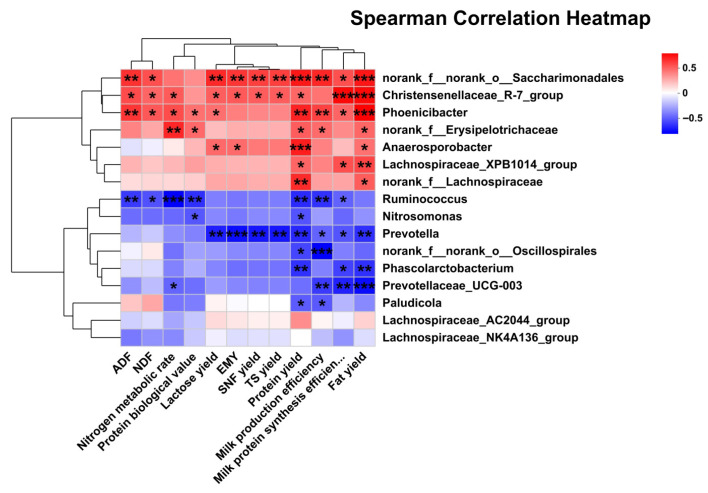
Pearson correlation analysis of differential bacteria genus with nutrient digestion and metabolism rate and lactation performance. Red indicates a positive correlation; blue indicates a negative correlation. * *p* < 0.05; ** *p* < 0.01; *** *p* < 0.001. Abbreviations: EMY = estimated milk yield.

**Figure 6:**
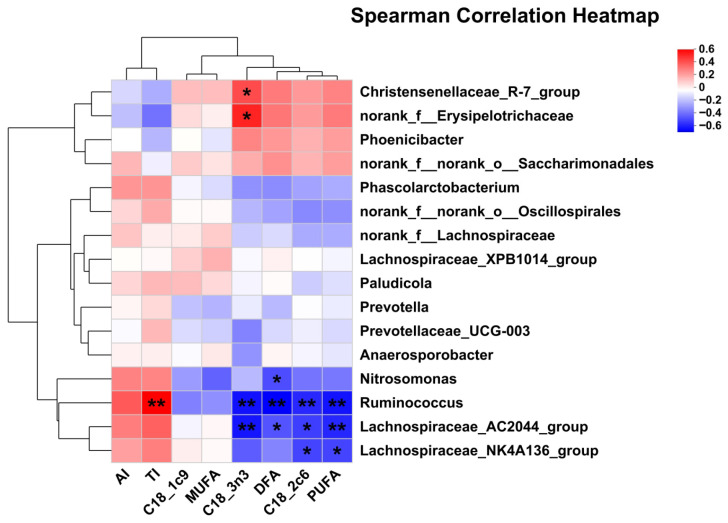
Pearson correlation analysis of differential bacteria genus and milk FA composition. Red indicates a positive correlation; blue indicates a negative correlation. * *p* < 0.05; ** *p* < 0.01. Abbreviations: AI = atherogenicity index (C12:0 + (4 × C14:0) + C16:0)/UFA; TI = thrombogenic index (C14:0 + C16:0 + C18:0)/[0.5 (MUFA) + 0.5 (n-6 PUFA) + 3 (n-3 PUFA) + (n-3 PUFA/n-6 PUFA)]; DFA = desirable fatty acid (C18:0 + UFA).

**Figure 7:**
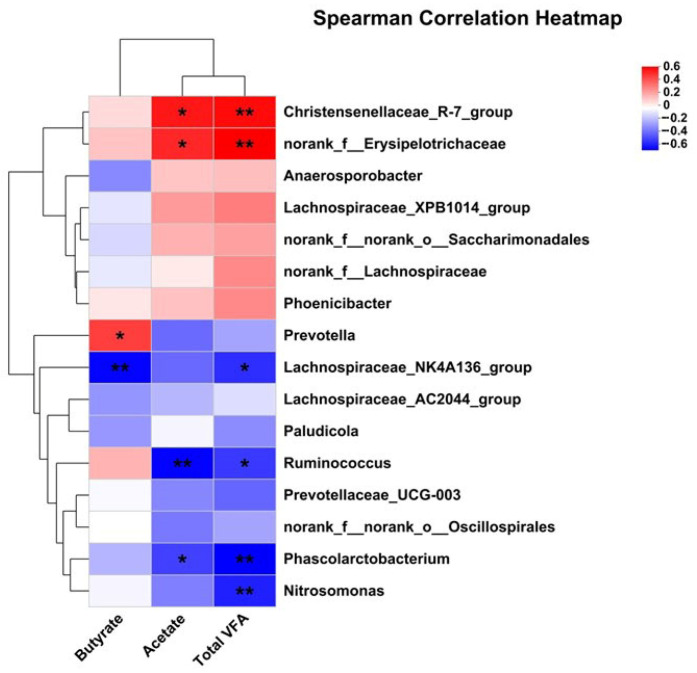
Pearson correlation analysis of differential bacteria genus and SCFA. Red indicates a positive correlation; blue indicates a negative correlation. * *p* < 0.05; ** *p* < 0.01.

A correlation heatmap based on Spearman’s correlation coefficient was generated to visualize the relationships among lactation performance, nutrient digestion and metabolism rates, milk FA composition, SCFA concentrations, and differentially abundant rectal bacteria (Figure 5, Figure 6 and Figure 7). The analysis between the significant differential bacteria and lactation performance revealed that the yields of TS and SNF were positively associated with *norank_f__norank_o__Saccharimonadales* and *Christensenellaceae_R-7_group*, but negatively associated with *Prevotella*. Lactose yield and EMY were positively associated with *norank_f__norank_o__Saccharimonadales*, *Christensenellaceae_R-7_group*, and *Anaerosporobacter*, but were negatively with *Prevotella*. Additionally, lactose yield was positively correlated with *Phoenicibacter*. Fat yield was positively associated with *Lachnospiraceae_XPB1014*, *norank_f__Lachnospiraceae*, *Anaerosporobacter*, *norank_f__norank_o__Saccharimonadales*, *Christensenellaceae_R-7_group*, *Phoenicibacter*, and *norank_f_Erysipelotrichaceae*, but was negatively with *Prevotella*, *Phascolarctobacterium*, and *Prevotellaceae_UCG-003*. Protein yield was positively associated with *Lachnospiraceae_XPB1014*, *norank_f__Lachnospiraceae*, *Anaerosporobacter*, *norank_f__norank_o__Saccharimonadales*, *Christensenellaceae_R-7_group*, *Phoenicibacter*, and *norank_f_Erysipelotrichaceae*, but was negatively with *Ruminococcus*, *Nitrosomonas*, *Prevotella*, *Phascolarctobacterium*, *norank_f_norank_o_Oscillospirales*, and *Paludicola*. Milk production efficiency was positively associated with *norank_f__norank_o__Saccharimonadales*, *Phoenicibacter* and *norank_f_Erysipelotrichaceae*, but was negatively with *Prevotella*, *Ruminococcus*, *Prevotellaceae_UCG-003*, *norank_f_norank_o_Oscillospirales*, and *Paludicola*. Milk protein synthesis efficiency was positively associated with *norank_f_norank_o_Saccharimonadales*, *Phoenicibacter*, *Christensenellaceae_R-7_group*, and *Lachnospiraceae_XPB1014*, but was negatively with *Ruminococcus*, *Prevotellaceae_UCG-003*, *Prevotella*, and *Phascolarctobacterium*.

In addition, the correlation analysis between significantly differential bacteria and nutrient digestion and metabolism rate revealed that ADF and NDF were positively associated with *norank_f_norank_o_Saccharimonadales*, *Christensenellaceae_R-7_group*, and *Phoenicibacter*, but were negatively with *Ruminococcus*. Protein biological value was positively associated with *Phoenicibacter* and *norank_f_Erysipelotrichaceae*, but was negatively with *Ruminococcus* and *Nitrosomonas*. Nitrogen metabolic rate was positively associated with *Phoenicibacter*, *Christensenellaceae_R-7_group*, and *norank_f_Erysipelotrichaceae*, but was negatively with *Ruminococcus* and *Prevotellaceae_UCG-003*.

The correlation analysis between significantly differential bacteria and milk FA composition revealed that C18:2c6 and PUFA were negatively associated with *Lachnospiraceae_NK4A136_group*, *Lachnospiraceae_AC2044_group*, and *Ruminococcus*. DFA was negatively associated with *Ruminococcus*, *Lachnospiraceae_AC2044_group*, and *Nitrosomonas*. C18:3n3 was negatively associated with *Lachnospiraceae_AC2044_group* and *Ruminococcus*, but was positively with *Christensenellaceae_R-7_group* and *norank_f_Erysipelotrichaceae*. TI was positively associated with *Ruminococcus*.

The correlation analysis between significantly differential bacteria and SCFA revealed that acetate was positively associated with *Christensenellaceae_R-7_group* and *norank_f_Erysipelotrichaceae*, but was negatively with *Ruminococcus* and *Phascolarctobacterium*. Butyrate was positively associated with *Prevotella*, but was negatively with *Lachnospiraceae_NK4A136_group*. Total VFA was positively associated with *Christensenellaceae_R-7_group* and *norank_f_Erysipelotrichaceae*, but was negatively with *Ruminococcus*, *Lachnospiraceae_NK4A136_group*, *Phascolarctobacterium*, and *Nitrosomonas*.

## 4. Discussion

### 4.1. Effects on Lactation Performance

Previous research on the impact of Se supplementation on lactation performance has predominantly focused on dairy cows [41], sheep [42], and sows [17], with limited studies specifically addressing lactating donkeys. Although Se is known to improve lactation in dairy cows [43,44], the optimal dosage and specific physiological responses in donkeys were previously unclear. This study found that supplementation with 0.3 mg Se/kg DM significantly increased milk yield, milk component yield, milk production efficiency, and milk protein synthesis efficiency. In contrast, supplementation with 0.5 mg Se/kg DM resulted in no improvement and even showed a potential inhibitory trend, suggesting that 0.3 mg Se/kg DM represents the optimal effective dose. Notably, the DMI of donkeys in the Se2 group remained unaffected. This indicates an increased efficiency of dietary nutrient utilization, which could explain the increased milk production. This finding was substantiated by the significant increase in the digestibility of NDF and ADF, as well as the BV and the N metabolic rate, observed in the Se2 group. From a physiological perspective, Se supplementation has been reported to strengthen mammary gland capillary distribution and increase vascular area [45], providing a histological basis for improved milk production. Furthermore, as shown in studies by Tong et al. [14] and Arshad et al. [46], Se supplementation improves lactation performance by improving mammary gland health, boosting antioxidant status, and reducing the incidence of mastitis. However, beyond a certain threshold, Se can exert pro-oxidant effects [47]. This may explain the inhibited lactation performance in the Se3 group of our study, which occurred despite a dose-dependent increase in plasma Se concentration. This potential mechanism, however, requires further validation.

Donkeys are monogastric herbivores that rely on hindgut fermentation. Their enlarged cecum and colon enable them to derive energy and nutrients from fibrous feeds through microbial fermentation [20]. The observed increase in milk yield may be closely related to the composition of the rectal microbiota. Since the rectal microbiota can, to a certain extent, reflect the microbial status in the cecum [22,23], and given that changes in the gut microbiota structure can affect cecal digestive function [48], the improvements in nutrient digestion and milk production observed in the Se2 group demonstrate the beneficial, dose-dependent effects of Se on gut microbiota. Se2 supplementation modulated specific beneficial bacterial genera. It significantly increased the abundance of *Christensenellaceae_R-7_group*, a probiotic commonly found in the intestinal tract and mucosa that is considered to be involved in amino acid and lipid metabolism [49]. Spearman correlation analysis revealed that *Christensenellaceae_R-7_group* was positively correlated with N metabolic rate, milk protein synthesis efficiency, protein yield, fat yield, acetate concentration, and the digestibility of ADF and NDF. Acetate serves as a primary substrate for milk fat synthesis [50]. LEfSe analysis also indicated significant enrichment of the probiotics *Lachnospiraceae_XPB1014_group* and *norank_f_Erysipelotrichaceae* in the Se2 group. Si et al. [51] had found that *Lachnospiraceae_XPB1014_group* was positively correlated with milk fat yield, which is consistent with this study. *Erysipelotrichaceae* predominantly participate in protein catabolism [52]. We found a positive correlation between *Erysipelotrichaceae* and the N metabolic rate, BV, protein yield, milk protein production efficiency, and milk production efficiency. Furthermore, the *Erysipelotrichaceae* also exhibited a positive correlation with acetate. Thus, the increased abundance of these bacteria may be the main reason for the improvements in nutrient digestibility and lactation performance observed in the Se2 group. In contrast, the abundance of these key beneficial genera was significantly lower in the high-dose Se3 group than in the Se2 group. This reduction may be a result of excessive blood Se concentrations.

### 4.2. Effects on Fatty Acid Composition of Milk

Supplementation with 0.3 mg Se/kg DM significantly increased the proportion of SFA in milk, notably elevating levels of short- and medium-chain fatty acids (SMCFA; C4:0, C6:0, C8:0, and C10:0). This elevation in SMCFA may be attributed to increased rectal concentrations of acetate and butyrate, which serve as substrates for the de novo synthesis of milk FAs in mammary epithelial cells [53]. The current study also confirmed that dietary supplementation with 0.3 and 0.5 mg Se/kg DM significantly increased the proportion of UFA in donkey milk. The underlying mechanism may be related to the Se-enhanced antioxidant capacity. This is supported by our plasma Se analysis, which showed a dose-dependent increase in concentration, and is consistent with our previous finding that these Se doses improved serum antioxidant function and reduced reactive oxygen species levels in lactating donkeys [14]. Furthermore, Yang et al. [54] reported that Se protects cell membranes from oxidative damage induced by reactive oxygen species and lipid hydroperoxides by inhibiting free radical generation. Supporting these findings, Ianni et al. [55] observed in dairy cows that dietary Se supplementation at 0.45 mg Se/kg DM significantly decreased the SFA content while increasing the concentration of C18:2c6 (a UFA) in milk.

Feed composition directly influences cecum fermentation and microbiota composition [56]. Consequently, dietary Se supplementation may increase UFA concentrations by modulating these processes in lactating donkeys. In this study, supplementation with 0.3 and 0.5 mg Se/kg DM increased the levels of C18:1c9, C18:2c6, and C18:3n3 in milk while decreasing the proportion of C18:0. This shift suggests that Se may inhibit the biohydrogenation (BH) process, leading to an accumulation of intermediate UFAs. Studies have found that *Prevotella* and *Ruminococus* may be involved in BH processes [57,58]. Our Spearman correlation analysis revealed that C18:2c6, C18:3n3, DFA, and PUFA were negatively correlated with *Ruminococus*, whereas TI showed a positive correlation. Furthermore, dietary supplementation with 0.3 mg Se/kg DM significantly increased the relative abundance of the *Christensenellaceae R-7 group*. This bacterial group can degrade plant cellulose and hemicellulose, converting them into SCFAs for host energy absorption [59]. Given that cellulose-hydrolyzing bacteria are involved in BH, we propose that Se may affect BH by increasing the abundance of *Christensenellaceae_R-7_group* and decreasing the abundance of *Ruminococus*—a causal relationship that warrants validation. The observed positive correlation between the abundance of the *Christensenellaceae_R-7_group* and milk C18:3n3 levels further suggests that this bacterial genus may play a pivotal role in C18:3n3 metabolism, potentially through its ability to convert C18:3n3 into beneficial metabolites for the host. Proposed metabolic pathways include the conversion of C18:3n3 to EPA and DHA, as well as the biosynthesis of health-promoting secondary metabolites via alternative biochemical routes. As C18:3n3 and its derivatives are known to confer anti-inflammatory and cardiovascular benefits [60], the *Christensenellaceae R-7 group* may indirectly improve host health by regulating C18:3n3 metabolism. Nevertheless, the precise molecular mechanisms require further elucidation.

P/S is an important indicator for assessing dietary nutritional value, with a recommended value of >0.45 considered desirable [61]. TI and AI are used to evaluate the fat quality of milk; lower values indicate a higher content of anti-atherosclerotic FA or a greater proportion of beneficial UFA [62]. In the present study, dietary supplementation with 0.3 and 0.5 mg Se/kg DM increased the P/S ratio, DFA, and the (C18:0 + C18:1)/C16:0 ratio, while decreasing AI and TI indices. These improvements enhance the nutritional value of donkey milk for human consumption, with the 0.5 mg Se/kg DM dose showing superior efficacy. MUFAs function beneficially in the regulation of plasma lipids and lipoproteins, resulting in decreased inflammation, oxidative stress, and coagulation, and improved glucose homeostasis and blood pressure control [63]. Our results showed that Se supplementation significantly increased MUFA. This suggests that the improvement in the antioxidant function of donkey milk previously observed with Se supplementation [14] may potentially be partially mediated by the increased MUFA proportion, though the specific underlying mechanism remains to be further explored and verified due to the complexity of the antioxidant regulatory network in milk.

In summary, dietary Se supplementation in lactating donkeys exhibits dose-dependent effects. A dose of 0.3 mg Se/kg DM was optimal, significantly improving lactation performance and milk yield. It also increased the proportions of beneficial FAs, such as C18:1c9, C18:2c6, C18:3n3, MUFA, PUFA, n-3 PUFA, and DFA; consequently, the overall FA profile of donkey milk was optimized. Although the 0.5 mg Se/kg DM dose showed no improvement and even a potential inhibitory effect on lactation performance, it was more effective at increasing the P/S ratio and reducing the AI and TI indices. Nonetheless, several limitations of this study should be acknowledged. First, the generalizability of our findings may be limited as they are based exclusively on lactating Dezhou donkeys. Furthermore, the study was constrained by a small sample size and a lack of functional analyses for the key bacterial genera identified. Consequently, the mechanistic insights remain associative and warrant further validation.

## 5. Conclusions

Dietary Se supplementation in lactating donkeys exhibits a dose-dependent effect: 0.3 mg Se/kg DM is the optimal level, as it significantly improves lactation performance and milk protein synthesis efficiency, increases the milk yield and proportions of FA, such as C18:1c9, C18:2c6, C18:3n3, MUFA, PUFA, n-3 PUFA, and DFA, and thereby optimizes FA profiles in donkey milk. The underlying mechanism may be related to promoting the digestion and metabolism of nutrients, as well as the enrichment of beneficial rectal bacteria such as *Christensenellaceae R-7 group*, *Lachnospiraceae XPB1014*, and *norank_f_Erysipelotrichaceae*. The 0.5 mg Se/kg DM dose showed no improvement or even a tendency to decrease lactation performance, but had better effects in increasing indices like the P/S ratio and decreasing AI and TI indices.

## Figures and Tables

**Table 1:** Composition and nutrient levels of basal diet (air-dry basis).

Items	Content
Feed Ingredients (% of DM)	
Millet straw	33.97
Alfalfa	23.55
Corn	15.19
Soybean meal	8.55
Corn silage	12.49
Distillers dried grains with solubles	1.80
Corn germ meal	1.80
Bran	0.90
NaCl	0.39
CaCO3	0.21
CaHPO4	0.66
Premix ^1^	0.50
Total	100.00
Nutrient composition (% of DM)	
DE (MJ/kg) ^2^	13.82
DM	87.21
CP	12.28
EE	2.12
NDF	47.87
ADF	28.30
Ca	1.12
P	0.36
Se, mg/kg DM	0.04

DE, digestible energy; DM, dry matter; CP, crude protein; EE, ether extract; NDF, neutral detergent fiber; ADF, acid detergent fiber; Ca, calcium; P, phosphorus. ^1^ Premix provided the following per kg of diet: vitamin A 6000 IU, vitamin D 1250 IU, vitamin E 15 IU, Fe 40 mg, Cu 8 mg, Zn 60 mg, Mn 60 mg, I 0.36 mg, Co 0.50 mg. ^2^ DE was a calculated value according to total energy and energy digestibility.

**Table 2:** Effects of Se supplementation on DMI, lactation performance, and SCC.

Items ^3^	Treatment				SEM ^2^	*p*-Value		
	CON^1^	Se1 ^1^	Se2 ^1^	Se3 ^1^		Treatment	Week	T × W
DMI (kg/day)	7.94	8.14	7.62	7.89	0.213	0.231	0.329	0.892
EMY (kg/day)	3.26 ^b^	3.23 ^b^	3.84 ^a^	3.50 ^b^	0.191	0.015	0.026	0.878
Milk production efficiency	0.23 ^b^	0.22 ^b^	0.28 ^a^	0.24 ^b^	0.014	0.001	<0.0001	0.842
Milk protein synthesis efficiency	0.75 ^b^	0.72 ^b^	0.88 ^a^	0.76 ^b^	0.045	0.002	<0.0001	0.913
Milk components								
Fat (%)	0.39	0.41	0.4	0.39	0.015	0.623	0.632	0.989
Protein (%)	1.83	1.82	1.82	1.81	0.017	0.869	<0.0001	0.999
Lactose (%)	7.11	7.11	7.12	7.12	0.021	0.918	<0.0001	0.404
SNF (%)	8.97	8.97	8.97	8.97	0.029	0.998	<0.001	0.841
TS (%)	9.38	9.37	9.36	9.38	0.028	0.955	<0.001	0.803
Milk components yield								
Fat (g/day)	11.98 ^b^	13.94 ^a^	15.54 ^a^	14.65 ^a^	0.736	0.008	0.220	0.640
Protein (g/day)	59.91 ^b^	59.78 ^b^	68.53 ^a^	65.05 ^ab^	3.880	0.063	0.002	0.965
Lactose (g/day)	227.58 ^b^	230.74 ^b^	270.77 ^a^	244.13 ^b^	13.911	0.008	0.001	0.725
SNF (g/day)	287.07 ^b^	291.82 ^b^	341.19 ^a^	306.64 ^b^	17.789	0.009	<0.001	0.763
TS (g/day)	299.64 ^b^	306.04 ^b^	355.80 ^a^	323.10 ^b^	18.594	0.012	0.001	0.831
SCC, ×1000 cells /mL	6.07 ^a^	5.26 ^ab^	4.23 ^b^	4.39 ^b^	0.431	0.019	<0.001	0.959

^a,b^ Means within a row with different letters are significantly different (*p* < 0.05). ^1^ CON = 0 mg of Se/kg DM; Se1 = 0.15 mg of Se/kg DM; Se2 = 0.3 mg of Se/kg DM; Se3 = 0.5 mg of Se/kg DM. ^2^ SEM = standard error of the mean. ^3^ DMI = dry matter intake; EMY = estimated milk yield; SNF = solids-not-fat; TS = total solids; SCC = somatic cell count.

**Table 3:** Effects of Se supplementation on apparent total-tract nutrient digestibility, energy, and protein metabolic ratio of lactating donkeys.

Items ^3^	CON ^1^	Se1 ^1^	Se2 ^1^	Se3 ^1^	SEM ^2^	*p*-Value
DM (%)	68.08	67.08	67.38	67.6	1.070	0.935
CP (%)	79.64	79.83	79.50	80.42	0.750	0.846
EE (%)	72.02	72.36	76.88	78.18	0.716	0.164
NDF (%)	38.49 ^b^	40.86 ^ab^	43.57 ^a^	44.53 ^a^	1.645	0.064
ADF (%)	32.58 ^b^	34.30 ^ab^	38.84 ^a^	39.57 ^a^	1.865	0.036
Energy metabolic rate (%)	67.92	68.07	70.56	69.58	0.260	0.199
BV (%)	79.29 ^b^	80.22 ^b^	86.54 ^a^	81.81 ^b^	0.609	0.019
Nitrogen metabolic rate (%)	63.67 ^b^	63.89 ^b^	71.12 ^a^	66.46 ^b^	0.594	0.012

^a,b^ Means within a row with different letters are significantly different (*p* < 0.05). ^1^ CON = 0 mg of Se/kg DM; Se1 = 0.15 mg of Se/kg DM; Se2 = 0.3 mg of Se/kg DM; Se3 = 0.5 mg of Se/kg DM. ^2^ SEM = standard error of the mean. ^3^ ADF = acid detergent fiber; CP = crude protein; DM = dry matter; EE = ether extract; NDF = neutral detergent fiber; BV = protein biological value.

**Table 4:** Effects of Se supplementation on plasma Se concentration and FA composition in lactating donkeys.

Items ^3^	CON ^1^	Se1 ^1^	Se2 ^1^	Se3 ^1^	SEM ^2^	*p*-Value
Plasma Se, ug/L	107.37 ^c^	157.20 ^b^	169.20 ^ab^	186.10 ^a^	6.733	<0.0001
SFA (%, TFA)						
C4:0	0.09 ^c^	0.09 ^c^	0.30 ^a^	0.12 ^b^	0.008	<0.0001
C6:0	0.06 ^c^	0.06 ^c^	0.19 ^a^	0.10 ^b^	0.005	<0.0001
C8:0	0.22 ^c^	0.21 ^c^	0.80 ^a^	0.40 ^b^	0.041	<0.0001
C10:0	0.14 ^b^	0.14 ^b^	0.05 ^c^	0.16 ^a^	0.006	<0.0001
C11:0	0.13 ^c^	0.06 ^d^	0.18 ^a^	0.16 ^b^	0.006	<0.0001
C12:0	0.06	0.06	0.04	0.04	0.008	0.133
C13:0	0.07 ^a^	0.07 ^a^	0.03 ^b^	0.07 ^a^	0.004	<0.0001
C14:0	0.38	0.38	0.39	0.40	0.010	0.235
C15:0	0.14	0.15	0.15	0.15	0.005	0.488
C16:0	15.81	15.77	15.34	16.57	0.343	0.108
C17:0	0.37 ^b^	0.41 ^a^	0.40 ^a^	0.40 ^a^	0.009	0.046
C18:0	18.83 ^a^	18.53 ^ab^	18.12 ^bc^	17.97 ^c^	0.172	0.007
C20:0	0.50 ^a^	0.43 ^b^	0.43 ^b^	0.43 ^b^	0.015	0.012
C21:0	0.04 ^c^	0.15 ^b^	0.07 ^c^	0.57 ^a^	0.022	<0.0001
C22:0	0.18 ^b^	0.20 ^b^	0.25 ^a^	0.19 ^b^	0.007	<0.0001
C23:0	0.55	0.61	0.58	0.61	0.042	0.715
C24:0	0.31	0.31	0.35	0.33	0.023	0.582
MUFA (%, TFA)						
C14:1	0.06	0.06	0.07	0.06	0.006	0.484
C15:1	0.03 ^b^	0.04 ^a^	0.04 ^a^	0.04 ^a^	0.002	<0.0001
C16:1	0.67	0.70	0.70	0.73	0.024	0.475
C17:1	0.17 ^c^	0.17 ^bc^	0.29 ^a^	0.20 ^b^	0.010	<0.0001
C18:1t9	0.08	0.09	0.08	0.08	0.006	0.508
C18:1c9	12.62	12.71	12.99	13.12	0.346	0.708
C20:1	0.42 ^a^	0.37 ^b^	0.36 ^b^	0.34 ^b^	0.013	0.005
C22:1	3.44 ^c^	4.04 ^b^	4.89 ^a^	4.11 ^b^	0.066	<0.0001
C24:1	0.59	0.58	0.65	0.66	0.046	0.471
n-6 PUFA (%, TFA)						
C18:2t6	0.07 ^a^	0.06 ^ab^	0.04 ^b^	0.07 ^a^	0.007	0.016
C18:2c6	41.87	42.65	42.75	43.26	0.536	0.351
C18:3n6	0.02 ^a^	0.02 ^a^	0.01 ^b^	0.02 ^a^	0.001	<0.001
C20:2n6	0.34	0.34	0.33	0.33	0.015	0.919
C20:3n6	0.13	0.14	0.13	0.13	0.010	0.783
C20:4n6	0.08	0.07	0.07	0.07	0.007	0.936
C22:2n6	0.07 ^b^	0.08 ^b^	0.14 ^a^	0.09 ^b^	0.015	0.009
n-3 PUFA (%, TFA)						
C18:3n3	0.74	0.76	0.76	0.75	0.040	0.993
C20:3n3	0.09 ^b^	0.09 ^b^	0.16 ^a^	0.10 ^b^	0.014	0.001
C20:5n3	0.04 ^b^	0.05 ^b^	0.07 ^a^	0.05 ^b^	0.005	0.002
C22:6n3	0.14 ^b^	0.15 ^ab^	0.16 ^ab^	0.17 ^a^	0.008	0.089
Sum and Ratio (%, TFA)						
SFA	38.35 ^a^	36.84 ^ab^	35.29 ^b^	35.62 ^b^	0.644	0.011
UFA	61.65 ^b^	63.16 ^ab^	64.71 ^a^	64.38 ^a^	0.644	0.011
MUFA	18.06 ^c^	18.75 ^bc^	20.08 ^a^	19.34 ^ab^	0.369	0.006
PUFA	43.59	44.41	44.63	45.04	0.534	0.293
n-3 PUFA	1.01	1.05	1.15	1.07	0.040	0.105
n-6 PUFA	42.58	43.36	43.48	43.97	0.536	0.345
n-3 LCPUFA	0.26 ^c^	0.29 ^bc^	0.39 ^a^	0.31 ^b^	0.012	<0.0001
n-6 LCPUFA	0.62	0.63	0.67	0.62	0.023	0.374
n-6/n-3	42.37	41.61	38.39	41.36	1.471	0.263
U/S	1.61 ^b^	1.72 ^ab^	1.84 ^a^	1.82 ^a^	0.049	0.013
P/S	1.14 ^b^	1.21 ^ab^	1.27 ^a^	1.27 ^a^	0.035	0.049
DFA	80.48 ^b^	81.69 ^ab^	82.83 ^a^	82.35 ^ab^	0.638	0.079
AI	0.28	0.27	0.26	0.28	0.007	0.107
TI	1.05 ^a^	1.01 ^a^	0.96 ^b^	1.00 ^ab^	0.016	0.006
(C18:0 + C18:1)/C16:0	2.00	1.99	2.05	1.89	0.047	0.141

^a,b,c,d^ Means within a row with different letters are significantly different (*p* < 0.05). ^1^ CON = 0 mg of Se/kg DM; Se1 = 0.15 mg of Se/kg DM; Se2 = 0.3 mg of Se/kg DM; Se3 = 0.5 mg of Se/kg DM. ^2^ SEM = standard error of the mean. ^3^ SFA = saturated fatty acid; UFA = unsaturated fatty acid; MUFA = monounsaturated fatty acid; PUFA = polyunsaturated fatty acids; DFA, desirable fatty acid (C18:0 + UFA); AI, atherogenicity index (C12:0 + (4 × C14:0) + C16:0) /UFA; TI, thrombogenic index (C14:0 + C16:0 + C18:0)/[0.5 (MUFA) + 0.5 (n-6 PUFA) + 3 (n-3 PUFA) + (n-3 PUFA/n-6 PUFA)].

**Table 5:** Effects of Se supplementation on FA composition in milk of lactating donkeys.

FA (%, TFA) ^3^	CON ^1^	Se1 ^1^	Se2 ^1^	Se3 ^1^	SEM ^2^	*p*-Value
SFA						
C4:0	0.18 ^b^	0.18 ^b^	0.20 ^a^	0.16 ^b^	0.006	0.003
C6:0	0.27 ^c^	0.31 ^b^	0.32 ^b^	0.44 ^a^	0.013	<0.0001
C8:0	5.16 ^b^	5.45 ^ab^	5.69 ^a^	5.01 ^b^	0.165	0.034
C10:0	12.22 ^bc^	13.42 ^ab^	13.69 ^a^	11.02 ^c^	0.448	0.001
C11:0	0.02 ^b^	0.02 ^b^	0.03 ^a^	0.03 ^a^	0.002	<0.0001
C12:0	9.51 ^a^	9.40 ^a^	9.35 ^a^	8.55 ^b^	0.224	0.023
C13:0	0.16 ^b^	0.21 ^a^	0.22 ^a^	0.20 ^a^	0.005	<0.0001
C14:0	6.79	6.59	6.53	6.49	0.269	0.867
C15:0	0.31 ^c^	0.39 ^a^	0.27 ^d^	0.35 ^b^	0.009	<0.0001
C16:0	22.21 ^a^	21.03 ^ab^	20.38 ^bc^	19.00 ^c^	0.529	0.002
C17:0	0.30	0.31	0.28	0.29	0.015	0.425
C18:0	1.77 ^a^	1.65 ^ab^	1.61 ^b^	1.60 ^b^	0.047	0.065
C20:0	0.05 ^a^	0.04 ^ab^	0.04 ^c^	0.04 ^b^	0.002	<0.001
C21:0	0.01	0.01	0.01	0.01	0.001	0.341
C22:0	0.02	0.01	0.01	0.01	0.001	0.115
C23:0	0.08	0.09	0.08	0.09	0.003	0.231
C24:0	0.01	0.01	0.01	0.01	0.001	0.113
MUFA						
C14:1	0.23 ^b^	0.23 ^b^	0.23 ^b^	0.29 ^a^	0.011	0.002
C15:1	0.01	0.01	0.01	0.01	0.001	0.145
C16:1	1.91 ^b^	2.46 ^a^	2.26 ^a^	2.21 ^a^	0.088	0.002
C17:1	0.01	0.01	0.01	0.01	0.002	0.779
C18:1t9	0.09 ^a^	0.04 ^c^	0.05 ^c^	0.08 ^b^	0.003	<0.0001
C18:1c9	18.29 ^b^	19.28 ^a^	19.16 ^a^	19.91 ^a^	0.278	0.004
C20:1	0.25 ^ab^	0.27 ^a^	0.24 ^ab^	0.23 ^b^	0.009	0.080
C22:1	0.09 ^a^	0.07 ^d^	0.08 ^c^	0.09 ^b^	0.001	<0.0001
C24:1	0.01	0.01	0.01	0.01	0.001	0.801
n-6 PUFA						
C18:2t6	0.01	0.01	0.01	0.01	0.001	0.771
C18:2c6	18.31 ^c^	18.58 ^bc^	19.60 ^b^	21.26 ^a^	0.407	<0.001
C18:3n6	0.02	0.02	0.02	0.02	0.002	0.732
C20:2n6	0.46 ^ab^	0.48 ^a^	0.42 ^b^	0.48 ^a^	0.012	0.018
C20:3n6	0.04	0.04	0.04	0.05	0.002	0.147
C20:4n6	0.01	0.01	0.01	0.01	0.001	0.731
C22:2n6	0.02 ^c^	0.03 ^b^	0.03 ^a^	0.03 ^a^	0.001	<0.0001
n-3 PUFA						
C18:3n3	3.38 ^b^	3.47 ^b^	3.62 ^b^	4.28 ^a^	0.090	<0.0001
C20:3n3	0.09 ^b^	0.10 ^a^	0.10 ^a^	0.10 ^a^	0.003	0.008
C20:5n3	0.01	0.01	0.01	0.01	0.001	0.152
C22:6n3	0.02	0.02	0.02	0.02	0.001	0.813
Sum and Ratio						
SFA	56.74 ^a^	54.84 ^b^	54.06 ^b^	50.90 ^c^	0.477	<0.0001
UFA	43.26 ^c^	45.16 ^b^	45.94 ^b^	49.10 ^a^	0.477	<0.0001
MUFA	20.88 ^b^	22.38 ^a^	22.05 ^a^	22.83 ^a^	0.305	0.001
PUFA	22.38 ^c^	22.78 ^bc^	23.89 ^b^	26.27 ^a^	0.448	<0.0001
n-3 PUFA	3.50 ^b^	3.60 ^b^	3.75 ^b^	4.41 ^a^	0.091	<0.0001
n-6 PUFA	18.88 ^b^	19.18 ^b^	20.14 ^b^	21.86 ^a^	0.413	<0.001
n-3 LCPUFA	0.12 ^b^	0.13 ^a^	0.13 ^a^	0.13 ^a^	0.003	0.006
n-6 LCPUFA	0.53 ^ab^	0.56 ^a^	0.51 ^b^	0.56 ^a^	0.013	0.018
n-6/n-3	5.42	5.33	5.38	4.98	0.142	0.130
U/S	0.76 ^c^	0.82 ^b^	0.85 ^b^	0.97 ^a^	0.017	<0.0001
P/S	0.39 ^c^	0.42 ^bc^	0.44 ^b^	0.52 ^a^	0.012	<0.0001
DFA	45.03 ^c^	46.81 ^b^	47.55 ^b^	50.71 ^a^	0.485	<0.0001
AI	1.36 ^a^	1.26 ^ab^	1.22 ^b^	1.09 ^c^	0.038	<0.001
TI	1.01 ^a^	0.92 ^b^	0.88 ^b^	0.76 ^c^	0.025	<0.0001
(C18:0 + C18:1)/C16:0	0.91 ^c^	1.00 ^b^	1.02 ^b^	1.14 ^a^	0.026	<0.0001

^a,b,c,d^ Means within a row with different letters are significantly different (*p* < 0.05). ^1^ CON = 0 mg of Se/kg DM; Se1 = 0.15 mg of Se/kg DM; Se2 = 0.3 mg of Se/kg DM; Se3 = 0.5 mg of Se/kg DM. ^2^ SEM = standard error of the mean. ^3^ SFA = saturated fatty acid; UFA = unsaturated fatty acid; MUFA = monounsaturated fatty acid; PUFA = polyunsaturated fatty acids; DFA, desirable fatty acid (C18:0 + UFA); AI, atherogenicity index (C12:0 + (4 × C14:0) + C16:0)/UFA; TI, thrombogenic index (C14:0 + C16:0 + C18:0)/[0.5 (MUFA) + 0.5 (n-6 PUFA) + 3 (n-3 PUFA) + (n-3 PUFA/n-6 PUFA)].

**Table 6:** Effects of Se supplementation on FA yield in milk of lactating donkeys.

FA (g/day) ^3^	CON1	Se1 ^1^	Se2 ^1^	Se3 ^1^	SEM ^2^	*p*-Value
SFA						
C4:0	0.56 ^bc^	0.61 ^b^	0.69 ^a^	0.50 ^c^	0.020	<0.0001
C6:0	0.86 ^c^	1.07 ^b^	1.12 ^b^	1.34 ^a^	0.046	<0.0001
C8:0	16.37 ^b^	18.67 ^a^	20.03 ^a^	15.46 ^b^	0.727	0.001
C10:0	38.89 ^b^	45.97 ^a^	48.13 ^a^	33.98 ^b^	1.817	<0.0001
C11:0	0.07 ^b^	0.06 ^b^	0.11 ^a^	0.10 ^a^	0.006	<0.0001
C12:0	30.34 ^a^	32.16 ^a^	32.90 ^a^	26.39 ^b^	1.236	0.005
C13:0	0.51 ^c^	0.74 ^a^	0.76 ^a^	0.63 ^b^	0.023	<0.0001
C14:0	21.64	22.58	22.97	20.00	1.076	0.236
C15:0	0.98 ^b^	1.32 ^a^	0.97 ^b^	1.09 ^b^	0.042	<0.0001
C16:0	70.75 ^a^	72.02 ^a^	71.95 ^a^	58.62 ^b^	2.846	0.006
C17:0	0.96	1.06	0.98	0.88	0.065	0.296
C18:0	5.64	5.66	5.67	4.96	0.232	0.109
C20:0	0.15	0.15	0.12	0.13	0.008	0.064
C21:0	0.007	0.02	0.01	0.01	0.005	0.305
C22:0	0.05	0.04	0.05	0.04	0.003	0.191
C23:0	0.27	0.29	0.29	0.28	0.011	0.229
C24:0	0.02	0.01	0.01	0.02	0.002	0.231
MUFA						
C14:1	0.73	0.79	0.82	0.88	0.042	0.101
C15:1	0.01	0.02	0.02	0.02	0.003	0.102
C16:1	6.07 ^b^	8.43 ^a^	8.00 ^a^	6.82 ^b^	0.401	0.001
C17:1	0.03	0.04	0.03	0.03	0.006	0.754
C18:1t9	0.28 ^a^	0.15 ^c^	0.16 ^c^	0.24 ^b^	0.010	<0.0001
C18:1c9	58.19 ^b^	66.10 ^a^	67.77 ^a^	61.42 ^ab^	2.419	0.039
C20:1	0.80 ^ab^	0.91 ^a^	0.87 ^a^	0.71 ^b^	0.044	0.022
C22:1	0.30 ^a^	0.25 ^b^	0.27 ^b^	0.27 ^b^	0.008	0.004
C24:1	0.04	0.04	0.04	0.04	0.003	0.725
n-6 PUFA						
C18:2t6	0.04	0.05	0.04	0.04	0.003	0.171
C18:2c6	58.37 ^b^	63.50 ^ab^	69.26 ^a^	65.76 ^ab^	2.635	0.050
C18:3n6	0.08	0.08	0.08	0.06	0.008	0.448
C20:2n6	1.47	1.63	1.50	1.47	0.066	0.268
C20:3n6	0.11 ^b^	0.14 ^a^	0.14 ^a^	0.14 ^a^	0.008	0.058
C20:4n6	0.01 ^c^	0.02 ^b^	0.03 ^a^	0.02 ^b^	0.001	<0.0001
C22:2n6	0.09 ^c^	0.11 ^b^	0.12 ^a^	0.11 ^b^	0.004	<0.0001
n-3 PUFA						
C18:3n3	10.76 ^b^	11.87 ^ab^	12.79 ^a^	13.23 ^a^	0.482	0.007
C20:3n3	0.28 ^b^	0.34 ^a^	0.36 ^a^	0.31 ^ab^	0.017	0.020
C20:5n3	0.03	0.03	0.04	0.03	0.003	0.363
C22:6n3	0.06	0.06	0.07	0.06	0.006	0.780
Sum and Ratio						
SFA	180.57 ^a^	187.64 ^a^	190.64 ^a^	157.05 ^b^	5.501	0.001
UFA	137.74 ^b^	154.57 ^ab^	162.38 ^a^	151.67 ^ab^	5.565	0.034
MUFA	66.45 ^b^	76.73 ^a^	77.97 ^a^	70.43 ^ab^	2.779	0.023
PUFA	71.29 ^b^	77.84 ^ab^	84.42 ^a^	81.24 ^a^	3.113	0.039
n-3 PUFA	11.12 ^b^	12.30 ^ab^	13.24 ^a^	13.64 ^a^	0.499	0.008
n-6 PUFA	60.17 ^b^	65.54 ^ab^	71.17 ^a^	67.60 ^ab^	2.699	0.056
n-3 LCPUFA	0.37 ^b^	0.43 ^a^	0.46 ^a^	0.41 ^ab^	0.021	0.029
n-6 LCPUFA	1.68	1.91	1.79	1.73	0.072	0.180
n-6/n-3	5.42	5.33	5.38	4.97	0.142	0.131
U/S	0.76 ^c^	0.82 ^b^	0.85 ^b^	0.97 ^a^	0.017	<0.0001
P/S	0.39 ^c^	0.42 ^bc^	0.44 ^b^	0.52 ^a^	0.012	<0.0001
DFA	143.39 ^b^	160.22 ^ab^	168.06 ^a^	156.63 ^ab^	5.737	0.041
AI	1.36 ^a^	1.26 ^ab^	1.22 ^b^	1.09 ^c^	0.038	<0.001
TI	1.01 ^a^	0.93 ^b^	0.88 ^b^	0.76 ^c^	0.025	<0.0001
(C18:0 + C18:1)/C16:0	0.91 ^c^	1.00 ^b^	1.02 ^b^	1.14 ^a^	0.026	<0.0001

^a,b,c^ Means within a row with different letters are significantly different (*p* < 0.05). ^1^ CON = 0 mg of Se/kg DM; Se1 = 0.15 mg of Se/kg DM; Se2 = 0.3 mg of Se/kg DM; Se3 = 0.5 mg of Se/kg DM. ^2^ SEM = standard error of the mean. ^3^ SFA = saturated fatty acid; UFA = unsaturated fatty acid; MUFA = monounsaturated fatty acid; PUFA = polyunsaturated fatty acids; DFA, desirable fatty acid (C18:0 + UFA); AI, atherogenicity index (C12:0 + (4 × C14:0) + C16:0)/UFA; TI, thrombogenic index (C14:0 + C16:0 + C18:0)/[0.5 (MUFA) + 0.5 (n-6 PUFA) + 3 (n-3 PUFA) + (n-3 PUFA/n-6 PUFA)].

**Table 7:** Effects of Se supplementation on SCFAs of rectal feces in lactating donkeys.

Items	CON ^1^	Se1 ^1^	Se2 ^1^	Se3 ^1^	SEM ^2^	*p*-Value
Acetate (mmol/L)	6.20 ^b^	6.32 ^b^	6.86 ^a^	6.88 ^a^	0.175	0.017
Propionate (mmol/L)	5.81	5.75	5.67	5.85	0.120	0.771
Butyrate (mmol/L)	0.81 ^c^	0.83 ^bc^	0.87 ^ab^	0.89 ^a^	0.017	0.014
Isobutyrate (mmol/L)	0.19	0.21	0.19	0.20	0.007	0.211
Valerate (mmol/L)	0.03	0.17	0.18	0.16	0.044	0.111
Isovalerate (mmol/L)	0.20 ^b^	0.24 ^a^	0.23 ^a^	0.23 ^a^	0.007	0.003
Acetate:Propionate ratio	1.07 ^b^	1.14 ^ab^	1.21 ^a^	1.18 ^a^	0.029	0.013
Total VFA (mmol/L)	13.26 ^b^	13.73 ^ab^	13.94 ^ab^	14.21 ^a^	0.228	0.046

^a,b,c^ Means within a row with different letters are significantly different (*p* < 0.05). ^1^ CON = 0 mg of Se/kg DM; Se1 = 0.15 mg of Se/kg DM; Se2 = 0.3 mg of Se/kg DM; Se3 = 0.5 mg of Se/kg DM. ^2^ SEM = standard error of the mean.

**Table 8:** Effects of Se supplementation on bacterial α-diversity in rectum of lactating donkeys.

Items	CON ^1^	Se1 ^1^	Se2 ^1^	Se3 ^1^	SEM ^2^	*p*-Value
Coverage	0.98	0.98	0.98	0.98	-	-
Sobs	1885.00	1913.67	1857.00	1965.40	46.161	0.573
Ace	2357.91	2444.62	2389.24	2436.86	54.377	0.754
Chao1	2363.21	2470.69	2381.10	2455.16	53.396	0.521
Shannon	5.96	5.93	5.88	5.96	0.094	0.964
Simpson	0.01	0.01	0.01	0.01	0.001	0.198

^1^ CON = 0 mg of Se/kg DM; Se1 = 0.15 mg of Se/kg DM; Se2 = 0.3 mg of Se/kg DM; Se3 = 0.5 mg of Se/kg DM. ^2^ SEM = standard error of the mean.

**Table 9:** Effects of Se supplementation on the composition of rectal bacteria at phyla level (more than 1% of total bacteria).

Phylum	Treatments				SEM ^2^	*p*-Value
	CON ^1^	Se1 ^1^	Se2 ^1^	Se3 ^1^		
Firmicutes	63.16	64.06	67.77	62.94	3.020	0.728
Bacteroidota	22.10	22.69	19.62	25.36	1.405	0.139
Verrucomicrobiota	3.51	4.92	5.14	4.99	0.983	0.706
Spirochaetota	5.87	3.23	4.60	4.33	0.720	0.198
Actinobacteriota	0.93 ^ab^	0.68 ^b^	1.37 ^a^	1.00 ^ab^	0.146	0.038

^a,b^ Means within a row with different letters are significantly different (*p* < 0.05). ^1^ CON = 0 mg of Se/kg DM; Se1 = 0.15 mg of Se/kg DM; Se2 = 0.3 mg of Se/kg DM; Se3 = 0.5 mg of Se/kg DM. ^2^ SEM = standard error of the mean.

## Data Availability

The raw 16S rRNA gene sequencing data have been deposited in the National Center for Biotechnology Information (NCBI) database under BioProject accession PRJNA1289364.

## Abbreviations

The following abbreviations are used in this manuscript:

AI	Atherogenicity index
AIA	Acid-insoluble ash
ATTD	Apparent total-tract digestibility
BH	Biohydrogenation
BV	Protein biological value
DFA	Desirable fatty acid
EE	Ether extract
EMY	Estimated milk yield
FA	Fatty acid
GE	Gross energy
MY	Milking yield
SCFA	Short-chain fatty acid
SCM	Solids-corrected milk
SMCFA	Short-and medium-chain fatty acid
TI	Thrombogenic index
Total VFA	Total volatile fatty acid
